# Tetra­aceto­nitrile­lithium tetra­iso­thio­cyanato­borate

**DOI:** 10.1107/S1600536813009082

**Published:** 2013-04-10

**Authors:** Jens Michael Breunig, Ulrich Wietelmann, Hans-Wolfram Lerner, Michael Bolte

**Affiliations:** a Institut für Anorganische und Analytische Chemie, Goethe-Universität Frankfurt, Max-von-Laue-Strasse 7, 60438 Frankfurt am Main, Germany; b Research & Development, Rockwood Lithium GmbH, Trakehner Str. 3, 60487 Frankfurt am Main, Germany

## Abstract

The crystal structure of the title salt, [Li(CH_3_CN)_4_][B(NCS)_4_], is composed of discrete cations and anions. Both the Li and B atoms show a tetra­hedral coordination by four equal ligands. The aceto­nitrile and iso­thio­cyanate ligands are linear. The bond angles at the B atom are close to the ideal tetra­hedral value [108.92 (18)–109.94 (16)°], but the bond angles at the Li atom show larger deviations [106.15 (17)–113.70 (17)°].

## Related literature

Our group is inter­ested in the synthesis of novel and improved electrolytes, namely borates with alkinyl or catecholate ligands, see: Lerner *et al.* (2007 ▶, 2012 ▶); Röder *et al.* (2008 ▶). For the preparation, see: Kleemann & Newman (1981 ▶).
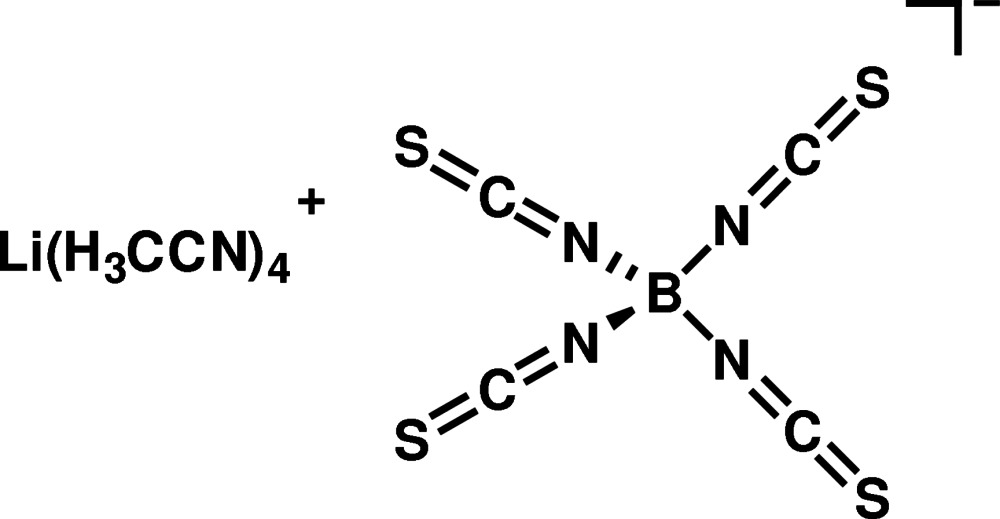


## Experimental

### 

#### Crystal data

[Li(C_2_H_3_N)_4_](C_4_BN_4_S_4_)
*M* *_r_* = 414.29
Monoclinic, 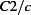

*a* = 21.219 (3) Å
*b* = 9.4756 (14) Å
*c* = 21.596 (4) Å
β = 92.845 (10)°
*V* = 4336.8 (12) Å^3^
*Z* = 8
Mo *K*α radiation
μ = 0.45 mm^−1^
*T* = 173 K
0.35 × 0.29 × 0.15 mm

#### Data collection

Stoe IPDS II two-circle diffractometer
Absorption correction: multi-scan (*X-AREA*; Stoe & Cie, 2001 ▶) *T*_min_ = 0.858, *T*_max_ = 0.936
24466 measured reflections
3816 independent reflections
2681 reflections with *I* > 2σ(*I*)
*R*_int_ = 0.074

#### Refinement

*R*[*F* ^2^ > 2σ(*F* ^2^)] = 0.038
*wR*(*F* ^2^) = 0.092
*S* = 0.97
3816 reflections
239 parameters
H-atom parameters constrained
Δρ_max_ = 0.18 e Å^−3^
Δρ_min_ = −0.25 e Å^−3^

### 

Data collection: *X-AREA* (Stoe & Cie, 2001 ▶); cell refinement: *X-AREA*; data reduction: *X-AREA*; program(s) used to solve structure: *SHELXS97* (Sheldrick, 2008 ▶); program(s) used to refine structure: *SHELXL97* (Sheldrick, 2008 ▶); molecular graphics: *XP* in *SHELXTL* (Sheldrick, 2008 ▶); software used to prepare material for publication: *SHELXL97* and *publCIF* (Westrip, 2010 ▶).

## Supplementary Material

Crystal structure: contains datablock(s) I, global. DOI: 10.1107/S1600536813009082/ng5320sup1.cif

Structure factors: contains datablock(s) I. DOI: 10.1107/S1600536813009082/ng5320Isup2.hkl

Additional supplementary materials:  crystallographic information; 3D view; checkCIF report

## Figures and Tables

**Fig. 1.**
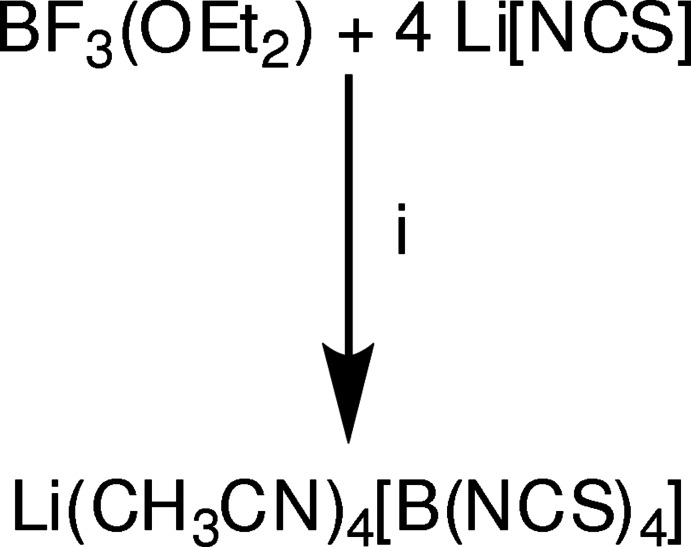
Synthesis of Li(CH3CN)4[B(NCS)4]. (i) -3LiF; in CH3CN.

**Fig. 2.**
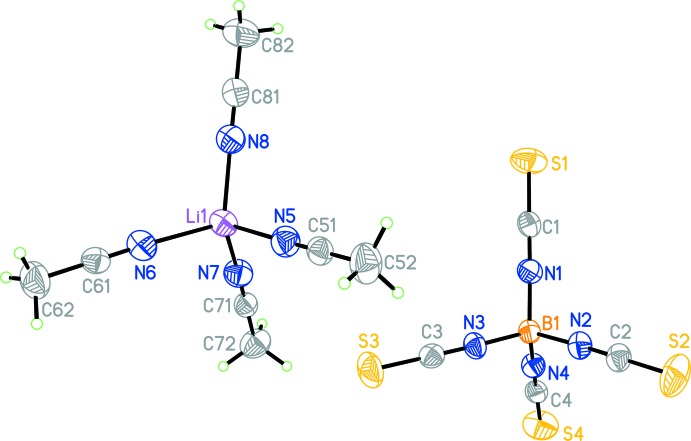
A perspective view of the title compound showing the atom-numbering scheme. Displacement ellipsoids are drawn at the 50% probability level and H atoms are shown as small spheres of arbitrary radii.

## supplementary crystallographic information

### Comment

Our group is interested in the synthesis of novel and improved electrolytes, namely, borates with alkinyl or catecholate ligands (Lerner *et al.*, 2007, 2012; Röder *et al.*, 2008). In the course of our investigations we synthesized the literature-reported borate Li[B(NCS)_4_] (Kleemann & Newman 1981) to compare its electrochemical properties with those of the borates which we have prepared. We were able to get crystals of this so far structurally uncharacterized borate Li(CH_3_CN)_4_[B(NCS)_4_]. The borate Li(CH_3_CN)_4_[B(NCS)_4_] was synthesized from BF_3_(OEt_2_) and Li[NCS], as shown in Figure 1.

The crystal structure of [Li(CH_3_CN)_4_]^+^[B(NCS)_4_]^-^ is composed of discrete cations and anions (Fig. 2). Both the Li and B centre, show a tetrahedral coordination by four equal ligands. The acetonitrile and the isothiocyanate ligands are linear. Whereas the bond angles at the boron centre [108.92 (18)° - 109.94 (16)°] are very close to the ideal tetrahedral value, the bond angles around the Li centre [106.15 (17)° - 113.70 (17)°] show larger deviations from the ideal value.

### Experimental

Li(CH_3_CN)_4_[B(NCS)_4_]: The borate Li(CH_3_CN)_4_[B(NCS)_4_] was prepared according to a literature procedure (Kleemann & Newman 1981). X-ray quality crystals of Li(CH_3_CN)_4_[B(NCS)_4_] were grown from an acetonitrile solution at room temperature.

### Refinement

All H atoms were located in difference Fourier maps. Nevertheless, they were geometrically positioned and refined using a riding model with C—H = 0.98 Å and with *U*_iso_(H) = 1.5*U*_eq_(C). The methyl groups were allowed to rotate but not to tip.

### Crystal data

**Table:**

[Li(C_2_H_3_N)_4_](C_4_BN_4_S_4_)	*F*(000) = 1696
*M**_r_* = 414.29	*D*_x_ = 1.269 Mg m^−^^3^
Monoclinic, *C*2/*c*	Mo *K*α radiation, λ = 0.71073 Å
Hall symbol: -C 2yc	Cell parameters from 13942 reflections
*a* = 21.219 (3) Å	θ = 3.6–26.8°
*b* = 9.4756 (14) Å	µ = 0.45 mm^−^^1^
*c* = 21.596 (4) Å	*T* = 173 K
β = 92.845 (10)°	Block, colourless
*V* = 4336.8 (12) Å^3^	0.35 × 0.29 × 0.15 mm
*Z* = 8	

### Data collection

**Table:**

Stoe IPDS II two-circle diffractometer	3816 independent reflections
Radiation source: Genix 3D IµS microfocus X-ray source	2681 reflections with *I* > 2σ(*I*)
Genix 3D multilayer optics monochromator	*R*_int_ = 0.074
ω scans	θ_max_ = 25.0°, θ_min_ = 3.6°
Absorption correction: multi-scan (*X-AREA*; Stoe & Cie, 2001)	*h* = −25→25
*T*_min_ = 0.858, *T*_max_ = 0.936	*k* = −11→11
24466 measured reflections	*l* = −25→25

### Refinement

**Table:**

Refinement on *F*^2^	Primary atom site location: structure-invariant direct methods
Least-squares matrix: full	Secondary atom site location: difference Fourier map
*R*[*F*^2^ > 2σ(*F*^2^)] = 0.038	Hydrogen site location: inferred from neighbouring sites
*wR*(*F*^2^) = 0.092	H-atom parameters constrained
*S* = 0.97	*w* = 1/[σ^2^(*F*_o_^2^) + (0.0478*P*)^2^] where *P* = (*F*_o_^2^ + 2*F*_c_^2^)/3
3816 reflections	(Δ/σ)_max_ = 0.032
239 parameters	Δρ_max_ = 0.18 e Å^−^^3^
0 restraints	Δρ_min_ = −0.25 e Å^−^^3^

### Special details

**Table:**

Geometry. All e.s.d.'s (except the e.s.d. in the dihedral angle between two l.s. planes) are estimated using the full covariance matrix. The cell e.s.d.'s are taken into account individually in the estimation of e.s.d.'s in distances, angles and torsion angles; correlations between e.s.d.'s in cell parameters are only used when they are defined by crystal symmetry. An approximate (isotropic) treatment of cell e.s.d.'s is used for estimating e.s.d.'s involving l.s. planes.
Refinement. Refinement of *F*^2^ against ALL reflections. The weighted *R*-factor *wR* and goodness of fit *S* are based on *F*^2^, conventional *R*-factors *R* are based on *F*, with *F* set to zero for negative *F*^2^. The threshold expression of *F*^2^ > σ(*F*^2^) is used only for calculating *R*-factors(gt) *etc*. and is not relevant to the choice of reflections for refinement. *R*-factors based on *F*^2^ are statistically about twice as large as those based on *F*, and *R*- factors based on ALL data will be even larger.

### Fractional atomic coordinates and isotropic or equivalent isotropic displacement parameters (Å^2^)

**Table:**

	*x*	*y*	*z*	*U*_iso_*/*U*_eq_	
B1	0.60133 (11)	0.2457 (2)	0.70356 (11)	0.0313 (4)	
N1	0.61748 (7)	0.15384 (18)	0.65017 (8)	0.0385 (4)	
C1	0.62999 (8)	0.0914 (2)	0.60595 (10)	0.0353 (4)	
S1	0.64743 (3)	0.00457 (7)	0.54619 (3)	0.06209 (19)	
N2	0.65695 (7)	0.33656 (17)	0.72237 (8)	0.0378 (4)	
C2	0.69788 (9)	0.4127 (2)	0.73635 (9)	0.0384 (5)	
S2	0.75423 (3)	0.51599 (7)	0.75594 (3)	0.0673 (2)	
N3	0.54597 (7)	0.33778 (16)	0.68497 (7)	0.0355 (4)	
C3	0.50357 (8)	0.4078 (2)	0.66964 (8)	0.0346 (4)	
S3	0.44491 (3)	0.50352 (7)	0.64826 (3)	0.06058 (19)	
N4	0.58433 (7)	0.15490 (16)	0.75811 (8)	0.0355 (4)	
C4	0.57138 (8)	0.0892 (2)	0.80105 (9)	0.0349 (4)	
S4	0.55383 (3)	−0.00256 (7)	0.85961 (3)	0.06261 (19)	
Li1	0.34824 (15)	0.4840 (3)	0.44482 (16)	0.0416 (8)	
N5	0.42234 (8)	0.6002 (2)	0.47394 (8)	0.0461 (4)	
C51	0.46315 (9)	0.6718 (2)	0.48727 (9)	0.0389 (5)	
C52	0.51502 (11)	0.7646 (3)	0.50381 (13)	0.0613 (7)	
H52A	0.5480	0.7536	0.4740	0.092*	
H52B	0.5323	0.7409	0.5455	0.092*	
H52C	0.5001	0.8625	0.5033	0.092*	
N6	0.27497 (8)	0.61203 (19)	0.42481 (8)	0.0464 (4)	
C61	0.23575 (9)	0.6908 (2)	0.41537 (9)	0.0386 (5)	
C62	0.18563 (11)	0.7913 (3)	0.40324 (13)	0.0620 (7)	
H62A	0.1819	0.8524	0.4395	0.093*	
H62B	0.1458	0.7410	0.3948	0.093*	
H62C	0.1952	0.8490	0.3672	0.093*	
N7	0.32273 (8)	0.36138 (19)	0.51545 (9)	0.0461 (4)	
C71	0.30814 (8)	0.3053 (2)	0.55915 (10)	0.0364 (4)	
C72	0.28959 (12)	0.2348 (3)	0.61477 (11)	0.0542 (6)	
H72A	0.3192	0.2594	0.6494	0.081*	
H72B	0.2901	0.1324	0.6083	0.081*	
H72C	0.2469	0.2647	0.6243	0.081*	
N8	0.37312 (8)	0.36597 (19)	0.37288 (9)	0.0444 (4)	
C81	0.38851 (9)	0.3023 (2)	0.33200 (11)	0.0398 (5)	
C82	0.40806 (13)	0.2207 (3)	0.27914 (13)	0.0646 (7)	
H82A	0.3716	0.1704	0.2603	0.097*	
H82B	0.4405	0.1525	0.2930	0.097*	
H82C	0.4253	0.2844	0.2485	0.097*	

### Atomic displacement parameters (Å^2^)

**Table:**

	*U*^11^	*U*^22^	*U*^33^	*U*^12^	*U*^13^	*U*^23^
B1	0.0281 (9)	0.0337 (10)	0.0321 (11)	0.0005 (8)	0.0013 (8)	0.0031 (9)
N1	0.0373 (8)	0.0408 (9)	0.0376 (10)	0.0027 (7)	0.0043 (7)	−0.0003 (8)
C1	0.0313 (9)	0.0317 (10)	0.0429 (12)	−0.0012 (7)	0.0029 (8)	0.0024 (9)
S1	0.0764 (4)	0.0521 (4)	0.0592 (4)	−0.0026 (3)	0.0184 (3)	−0.0211 (3)
N2	0.0326 (8)	0.0397 (9)	0.0408 (10)	−0.0015 (7)	0.0002 (7)	0.0026 (7)
C2	0.0355 (10)	0.0433 (11)	0.0362 (11)	0.0007 (9)	−0.0006 (8)	0.0079 (9)
S2	0.0574 (4)	0.0733 (4)	0.0692 (4)	−0.0323 (3)	−0.0161 (3)	0.0098 (3)
N3	0.0304 (8)	0.0381 (9)	0.0379 (9)	0.0059 (7)	0.0014 (7)	0.0063 (7)
C3	0.0363 (10)	0.0379 (10)	0.0299 (10)	−0.0035 (8)	0.0042 (8)	0.0033 (8)
S3	0.0462 (3)	0.0712 (4)	0.0640 (4)	0.0258 (3)	−0.0017 (3)	0.0165 (3)
N4	0.0356 (8)	0.0364 (8)	0.0345 (9)	0.0019 (7)	0.0018 (7)	0.0053 (8)
C4	0.0346 (9)	0.0342 (10)	0.0358 (11)	0.0057 (8)	0.0012 (8)	−0.0002 (9)
S4	0.0843 (4)	0.0581 (4)	0.0470 (4)	0.0039 (3)	0.0194 (3)	0.0217 (3)
Li1	0.0411 (16)	0.0402 (18)	0.0440 (19)	0.0036 (15)	0.0058 (14)	−0.0016 (16)
N5	0.0415 (9)	0.0511 (11)	0.0457 (11)	−0.0003 (8)	0.0024 (8)	0.0040 (8)
C51	0.0370 (10)	0.0427 (11)	0.0369 (11)	0.0075 (9)	0.0026 (8)	0.0065 (9)
C52	0.0456 (14)	0.0583 (15)	0.0791 (19)	−0.0065 (10)	−0.0060 (13)	−0.0049 (13)
N6	0.0430 (9)	0.0462 (10)	0.0503 (11)	0.0022 (8)	0.0063 (8)	0.0010 (8)
C61	0.0400 (10)	0.0378 (11)	0.0382 (11)	−0.0022 (9)	0.0047 (8)	−0.0038 (9)
C62	0.0579 (14)	0.0531 (14)	0.0741 (18)	0.0181 (11)	−0.0067 (13)	−0.0046 (13)
N7	0.0463 (9)	0.0457 (10)	0.0464 (11)	−0.0019 (8)	0.0051 (8)	0.0002 (9)
C71	0.0320 (9)	0.0353 (10)	0.0415 (12)	−0.0019 (8)	−0.0015 (8)	−0.0067 (9)
C72	0.0594 (14)	0.0597 (15)	0.0436 (13)	−0.0108 (11)	0.0026 (11)	0.0079 (11)
N8	0.0460 (9)	0.0428 (10)	0.0445 (11)	0.0013 (8)	0.0043 (8)	0.0028 (9)
C81	0.0384 (10)	0.0357 (11)	0.0454 (13)	−0.0015 (8)	0.0028 (9)	0.0067 (10)
C82	0.0736 (17)	0.0627 (16)	0.0587 (16)	0.0072 (13)	0.0154 (13)	−0.0113 (13)

### Geometric parameters (Å, º)

**Table:**

B1—N1	1.498 (3)	C52—H52A	0.9800
B1—N2	1.501 (3)	C52—H52B	0.9800
B1—N3	1.502 (2)	C52—H52C	0.9800
B1—N4	1.516 (3)	N6—C61	1.129 (2)
N1—C1	1.165 (3)	C61—C62	1.442 (3)
C1—S1	1.589 (2)	C62—H62A	0.9800
N2—C2	1.158 (2)	C62—H62B	0.9800
C2—S2	1.586 (2)	C62—H62C	0.9800
N3—C3	1.153 (2)	N7—C71	1.139 (3)
C3—S3	1.5900 (19)	C71—C72	1.446 (3)
N4—C4	1.161 (2)	C72—H72A	0.9800
C4—S4	1.594 (2)	C72—H72B	0.9800
Li1—N5	1.995 (4)	C72—H72C	0.9800
Li1—N6	2.002 (4)	N8—C81	1.131 (3)
Li1—N8	2.006 (4)	C81—C82	1.456 (4)
Li1—N7	2.013 (4)	C82—H82A	0.9800
N5—C51	1.126 (2)	C82—H82B	0.9800
C51—C52	1.440 (3)	C82—H82C	0.9800
			
N1—B1—N2	109.56 (18)	H52A—C52—H52C	109.5
N1—B1—N3	109.73 (15)	H52B—C52—H52C	109.5
N2—B1—N3	109.44 (15)	C61—N6—Li1	175.7 (2)
N1—B1—N4	109.94 (16)	N6—C61—C62	179.9 (3)
N2—B1—N4	109.24 (16)	C61—C62—H62A	109.5
N3—B1—N4	108.92 (18)	C61—C62—H62B	109.5
C1—N1—B1	174.95 (19)	H62A—C62—H62B	109.5
N1—C1—S1	179.26 (19)	C61—C62—H62C	109.5
C2—N2—B1	176.46 (18)	H62A—C62—H62C	109.5
N2—C2—S2	179.5 (2)	H62B—C62—H62C	109.5
C3—N3—B1	178.8 (2)	C71—N7—Li1	172.5 (2)
N3—C3—S3	179.6 (2)	N7—C71—C72	179.7 (3)
C4—N4—B1	177.82 (19)	C71—C72—H72A	109.5
N4—C4—S4	179.3 (2)	C71—C72—H72B	109.5
N5—Li1—N6	108.97 (17)	H72A—C72—H72B	109.5
N5—Li1—N8	108.54 (17)	C71—C72—H72C	109.5
N6—Li1—N8	113.70 (17)	H72A—C72—H72C	109.5
N5—Li1—N7	108.48 (17)	H72B—C72—H72C	109.5
N6—Li1—N7	106.15 (17)	C81—N8—Li1	178.0 (2)
N8—Li1—N7	110.86 (17)	N8—C81—C82	179.7 (3)
C51—N5—Li1	175.4 (2)	C81—C82—H82A	109.5
N5—C51—C52	179.3 (2)	C81—C82—H82B	109.5
C51—C52—H52A	109.5	H82A—C82—H82B	109.5
C51—C52—H52B	109.5	C81—C82—H82C	109.5
H52A—C52—H52B	109.5	H82A—C82—H82C	109.5
C51—C52—H52C	109.5	H82B—C82—H82C	109.5

## References

[bb1] Kleemann, L. P. & Newman, G. H. (1981). US Patent No. 4 279 976.

[bb2] Lerner, H.-W., Röder, J., Vitze, H., Bolte, M., Wagner, M. & Wietelmann, U. (2007). Ger. Patent No. 10 2007 047 812 A1.

[bb3] Lerner, H.-W., Röder, J., Vitze, H., Bolte, M., Wagner, M. & Wietelmann, U. (2012). US Patent No. 8 222 457.

[bb4] Röder, J., Wietelmann, U., Vitze, H., Bolte, M., Lerner, H.-W. & Wagner, M. (2008). Ger. Patent No. 10 2008 041 812 A1.

[bb5] Sheldrick, G. M. (2008). *Acta Cryst.* A**64**, 112–122. 10.1107/S0108767307043930 18156677

[bb6] Stoe & Cie (2001). *X-AREA* Stoe & Cie, Darmstadt, Germany.

[bb7] Westrip, S. P. (2010). *J. Appl. Cryst.* **43**, 920–925.

